# The *pros* and *cons* of ubiquitination on the formation of protein condensates: Ubiquitination on protein condensates

**DOI:** 10.3724/abbs.2023096

**Published:** 2023-06-09

**Authors:** Xue-Ni Hou, Chun Tang

**Affiliations:** 1 Beijing National Laboratory for Molecular Sciences College of Chemistry and Molecular Engineering Peking University Beijing 100871 China; 2 Center for Quantitate Biology PKU-Tsinghua Center for Life Science Academy for Advanced Interdisciplinary Studies Peking University Beijing 100871 China

**Keywords:** ubiquitination, phase separation, stress granule, polyubiquitin chain, post-translational modification

## Abstract

Ubiquitination, a post-translational modification that attaches one or more ubiquitin (Ub) molecules to another protein, plays a crucial role in the phase-separation processes. Ubiquitination can modulate the formation of membrane-less organelles in two ways. First, a scaffold protein drives phase separation, and Ub is recruited to the condensates. Second, Ub actively phase-separates through the interactions with other proteins. Thus, the role of ubiquitination and the resulting polyUb chains ranges from bystanders to active participants in phase separation. Moreover, long polyUb chains may be the primary driving force for phase separation. We further discuss that the different roles can be determined by the lengths and linkages of polyUb chains which provide preorganized and multivalent binding platforms for other client proteins. Together, ubiquitination adds a new layer of regulation for the flow of material and information upon cellular compartmentalization of proteins.

## Introduction

A cell has many organelles and compartments to perform location-specific functions. Membrane-less organelles (MLOs) are formed upon the phase separation of biomolecules [ 1– 3] . Among them, nuclear speckles, nucleolus, promyelocytic leukemia (PML) protein bodies, and Cajal bodies in the nucleus, and stress granules (SGs), processing bodies (P-bodies), and signaling puncta in the cytoplasm are well noted [ 1, 4– 11] .

Phase separation and phase transition have been extensively studied in polymer science but have recently garnered increasing attention for a better understanding of biological processes [ 12– 15] . Phase separation is mainly driven by minimizing the global free energy of the system, though the newly formed phases can be kinetically trapped [ 16, 17] . Phase separation in the living system usually involves multiple components whose properties and concentrations are metabolically regulated [18]. Many factors have been shown to modulate phase separation, such as temperature, hydrostatic pressure, osmolarity, pH, salt type and concentration, and macromolecular crowding [ 19– 21] . In addition, post-translational modifications (PTMs) have been shown to change the chemical properties of key interacting residues of the phase-separating proteins, thus shifting the equilibrium of coacervation [ 7, 22– 30] .

Among the various modifications, ubiquitination and ubiquitin-like (UBL) modifications are particularly complex, as they conjugate the phase-separating proteins with another protein rather than small chemical moieties. In this review, we aim to provide, from both biological and chemical perspectives, insights into how ubiquitination regulates protein phase separation.

**Figure 1:**
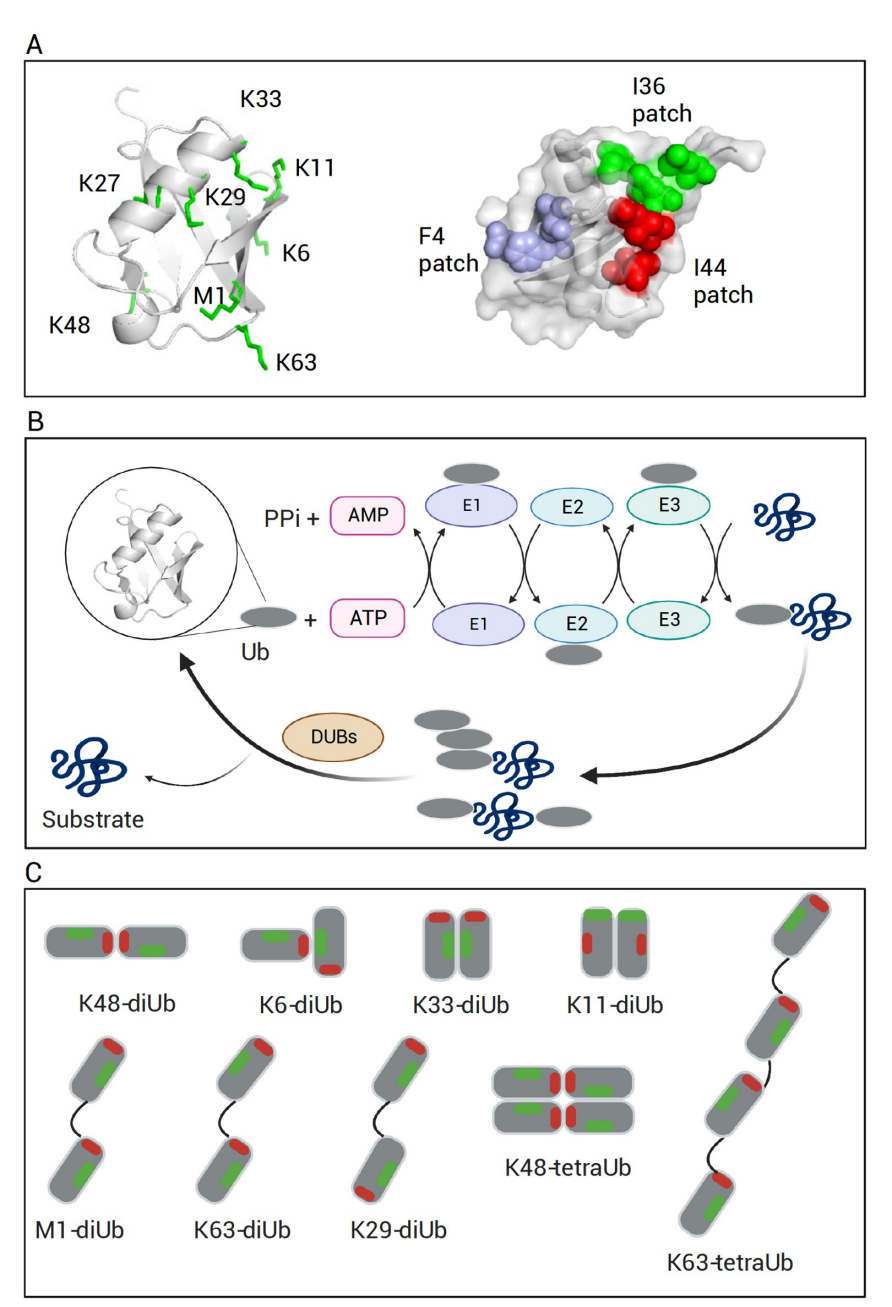
Figure 1 Ub exists as covalently linked polymers (A) Ub is shown as cartoon, on which eight residues could be covalently linked to another Ub are shown in sticks (green) (left); The surface of Ub is shown with hydrophobic patches colored: F4 patch (light blue), I36 patch (green) and I44 patch (red); (B) The synthesis of a polyUb chain and conjugation to a substrate protein is accomplished with a cascade of E1, E2, and E3 enzymes, and the modification is removed with deubiquitinases (DUBs). (C) A polyUb is conformationally heterogeneous, which depends on the covalent Ub linkages and noncovalent Ub-Ub interactions. A covalently linked di-ubiquitin (diUb) can exhibit different relative orientations between the two Ub subunits. Schematic representations of known diUb structures are shown (K48-diUb: 3m3j [34]; K6-diUb:2xk5 [35]; K33-diUb:4xyz [36]; K11-diUb: 2xew [37]; M1-diUb: 2w9n [38]; K63-diUb: 2jf5 [38]; K29-diUb: 4s22 [39]). I44 patches (including residues L8, I44, H68, and V70, shown in red) and I36 patches (including residues I36, L71, and L73, shown in green) are indicated, which are known interacting surfaces. The models of K48-tetraUb and K63-tetraUb are based on references [ 40, 41] . Generated with BioRender.com.

Ubiquitin (Ub) is a 76-residue protein with a molecular weight of ~8500 Da and is highly conserved over eukaryotes. It adopts a compact β-grasp fold with a flexible C-terminal tail. Ub noncovalently interacts with thousands of partner proteins via several motifs on its surface, separately or simultaneously. The interacting surfaces include the I44 hydrophobic patch (usually comprising residues I44, L8, H68, and V70), the I36 hydrophobic patch (comprising residues I36, L71, and L73), and the F4 patch (comprising residues F4, Q2, and T12) [ 31, 32] ( Figure 1A). On the other hand, Ub covalently modifies other proteins by forming an isopeptide bond between the C-terminus of Ub and the ε-amine of a lysine residue of another protein, either a substrate protein or another Ub [33]. The process is known as ubiquitination.

Ubiquitination is mediated by a cascade of enzymes in three steps ( Figure 1B). First, the activating enzyme E1 employs one ATP molecule to activate Ub by forming a high-energy thioester bond between the C-terminus of Ub and the catalytic cysteine residue of E1 (E1~Ub). Subsequently, E1 binds to another ATP molecule and transfers E1~Ub to the catalytic cysteine residue of the conjugating enzyme E2, affording the E2~Ub adduct. Finally, E3 ligase recruits E2~Ub, and the Ub is further transferred to a specific substrate in a direct or indirect manner, depending on the type of E3 ligase [ 42– 45] .

E3 ligases can be classified into three families: really interesting new gene (RING) E3s, homologous to E6-AP C-terminus (HECT) E3s, and RING-between-RING (RBR) E3s. During the final transferring step, RING E3 simultaneously interacts with the E2 and substrate protein, allowing a direct transfer of Ub to the substrate protein. In comparison, both HECT E3 and RBR E3 form an intermediate thioester-bonded complex with Ub before the subsequent conjugation to the substrate protein [ 46– 48] . Reciprocally, Ub modifications at substrate proteins can be removed by a superfamily of deubiquitinases (DUBs). So far, it is known that the human genome encodes two E1s, around 40 E2s, over 600 E3s, and no less than 100 DUBs [ 49– 53] .

A salient feature of ubiquitination is its high versatility in the formation of polyUb (polyUb) chains, generating a multitude of covalent Ub linkages. The amine group can arise from one of the lysine sidechains (K6, K11, K27, K29, K33, K48, and K63) or the N-terminus of another Ub ( Figure 1A). For the M1-linked, linear polyUb chain, two Ub subunits are concatenated, with a peptide bond formed between Ubs. The M1-linked chain can either be the direct gene expression product, i.e., *ubc* and *ubb* genes, or catalyzed with the LUBAC complex [ 54– 56] . Moreover, different Ub linkages can mix and match, affording mixed- or branched-polyUb chains [ 57, 58] .

Ub monomers can noncovalently interact with each other—the phenomenological binding affinity is in the millimolar range [59]. When two Ubs are covalently linked through a Ub linkage, the effective concentration for the noncovalent interaction becomes much higher, and a polyUb chain would adhere to the sticker-and-spacer model for phase separation [60]. The interactions may occur between the immediately adjacent or distant Ub subunits in a polyUb chain. On the other hand, a polyUb chain interacts with other proteins, which further promotes phase separation [ 61– 69] .

In this review, we propose that Ub signaling plays a critical role in a broad spectrum of cellular processes through mechanisms related to phase separation. First, we discuss the role of ubiquitination in regulating the SG dynamics and driving the signal puncta formation involved in protein degradation and other signaling pathways. Moreover, we correlate the chain linkages and lengths to their biological functions and propose the underlying physical principles for the disparate regulatory effects of ubiquitination on phase separation.

## Biological Evidence for Ubiquitination in the Regulation of Phase Separation

### Stress granule dynamics

SGs are molecular assemblies that appear in the cytoplasm in response to various stimuli [70]. SGs are dynamic, as these condensates form rapidly under stress conditions and conversely, disassemble rapidly after the relief of the stress. The exact compositions of SGs vary depending on the cellular context, the stress factor, and the signaling pathway involved [71]. The SG-causing stresses include oxidative stress, hypoxia, osmotic stress, heat shock stress, glucose starvation, sodium arsenite treatment, UV radiation, endoplasmic reticulum stress, viral infection, knockout of translation initiation factors, and overexpression of specific RNA-binding proteins (RBPs) [ 7, 71– 77] .

**Figure 2:**
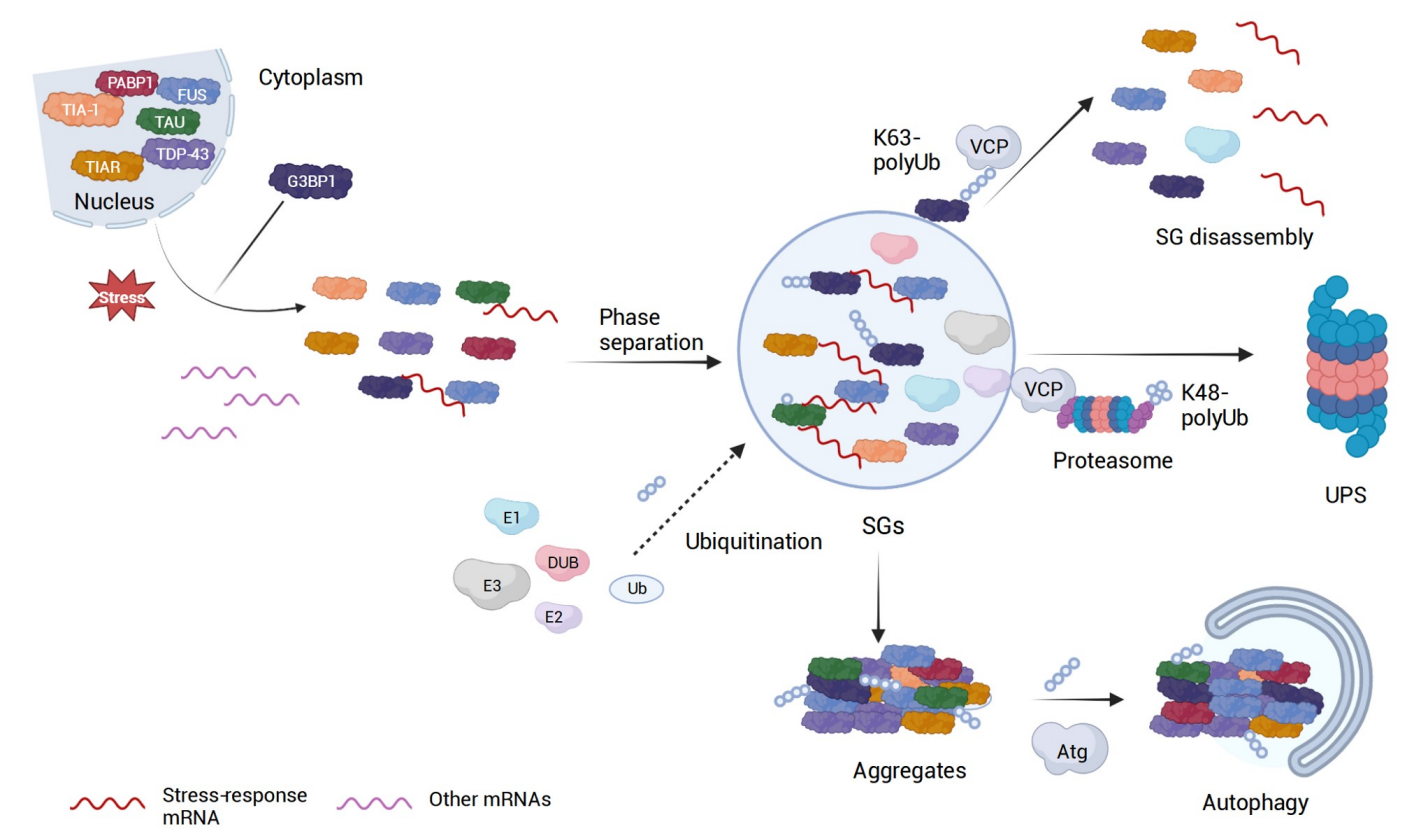
Figure 2 A schematic illustration of stress granule dynamics The SGs, a phase-separated membrane-less organelle, form under certain cellular stress conditions, including RNAs and RNA-binding proteins, such as G3BP1, TDP-43, and TIA-1. It is unclear whether the initial assembly of SGs is ubiquitination-dependent (dashed line), while the disassembly generally involves Ub modification. For example, K63-linkage ubiquitination of G3BP1 promotes its interaction with VCP, as required for the SG disassembly. Moreover, VCP recruits 26S proteasome to facilitate the clearance through the Ub-proteasome system (UPS), in which K48-linkage ubiquitination is involved. Prolonged stress or disruption of proteostasis can lead to the formation of aberrant SGs and insoluble aggregates, whose ultimate removal is mediated by autophagy, a process in which K63-linkage ubiquitination is involved. Generated with BioRender.com.

RBPs detected in the SGs include Ras GTPase-activating protein (SH3 domain)-binding proteins (G3BPs), polyadenylated [poly(A) ^+^] mRNA binding protein I (PABP1), T-cell internal antigen 1(TIA-1), TIA-1-related protein (TIAR), survival motor neuron protein (SMN), FUS, fragile X mental retardation protein 1 (FMRP1), DEAD box protein 1(DDX1), TDP-43, and ataxin-2 [ 78– 88] . Intriguingly (but not surprisingly), most of these RBPs can be modified by ubiquitin. Moreover, various E3 ligases, such as histone E3 ligase 2 (Hel2), the anaphase-promoting complex (APC), and tripartite motif protein family members (TRIM32, TRIM63, TRIM5α), DUBs, such as Ub-specific processing proteases (USP5, USP10, USP13) and the ovarian tumor (OTU) domain-containing protein 4 (OTUD4), and Ub-binding proteins (UBPs), such as Ub-associated protein 2-like (UBAP2L), UBQLN2, and histone deacetylase 6 (HDAC6) have been identified in the SGs [ 75, 89– 97] . The appearance of all these Ub-related proteins indicates a vital role of ubiquitination in the SG lifecycle. Figure 2 illustrates the potential regulatory effects of ubiquitination on SG dynamics, including the formation, disassembly, and clearance of the SGs, with a detailed explanation given below.

Firstly, the potential roles of Ub modifications and Ub binding in SG assembly have been uncovered by means of pharmacological inhibition. Acute inhibition of protein ubiquitination by the E1 inhibitor TAK243 does not prevent the SG formation in response to heat shock or arsenite, suggesting that active protein ubiquitination is not essential to SG formation. On the other hand, the authors did observe that unanchored Ub chains are detectable in the SGs that are important for SG disassembly [ 98, 99] . This finding is consistent with another observation that ubiquilin-2 (UBQLN2), a protein found in the SGs, forms liquid droplets both *in* *vivo* and *in* *vitro*, which could be disrupted with the addition of free Ub (Ub monomers) through the binding to C-terminal Ub-associating (UBA) domain of UBQLN2 [97]. Interestingly, the roles of distinct polyUb chains in regulating phase separation can differ [66], as we discuss below.

Besides UBQLN2, several other Ub-binding proteins (UBPs) have been reported to participate in the formation of SGs, and at the same time, could be modulated by ubiquitination. UBAP2L, composed of an N-terminal UBA domain and a C-terminal intrinsically disordered region (IDR), is reported to regulate SG dynamics positively [ 100, 101] . *UBAP2L* knockout strongly reduces the size of the granules. UBP2AL acts upstream of G3BP1 and facilitates the formation of the G3BP1 core [96]. Overexpression of the full-length UBAP2L in *UBAP2L*-KO cells could efficiently restore the assembly of SGs. In contrast, overexpression of the UBAP2L mutant with the deletion of the UBA domain only partially recovers the assembly, indicating the functional importance of the UBAP2L UBA domain for the formation of SGs [ 100, 102] . At a molecular level, the UBAP2L UBA domain may interact with unanchored Ub monomers, polyUb chains, or ubiquitinated-substrate proteins. Thus, Ub-UBA interactions promote phase separation in this case.

The cytoplasmic deacetylase HDAC6 has also been reported to localize in SGs through its interactions with G3BP1 or TIA-1 [ 103, 104] . HDAC6 contains a C-terminal Zinc-finger UBP (ZnF-UBP) domain and has a binding preference for K63-linked polyUb. Under stress conditions, such as arsenite-induced oxidative stress, UV irradiation, mitochondrial stress, or heat shock, deletion of the ZnF-UBP domain impairs Ub colocalization and the SG formation, suggesting that other ubiquitinated proteins are likely recruited through the interactions with HDAC6 [103]. However, in a separate study, the ZnF-UBP domain deletion does not affect the SG assembly in response to coxsackievirus A16 (CA16) infection, even though fewer Ub proteins are found localized in the SGs [105]. These observations are consistent with the results obtained with the E1 inhibitor in which SG formation does not critically depend on protein ubiquitination, despite a prevalent presence of Ub in the SGs.

Recent studies have shown that G3BP1 is also modified by the Ub chains, mostly K63-linkage, when SGs are assembled and formed in response to heat shock [ 99, 106] . Furthermore, the authors found that the N-terminal NTF2-like (NTF2L) domain of G3BP1 is ubiquitinated preferentially upon heat shock for the subsequent interaction with valosin-containing protein (VCP), also known as Ub-selective p97. The G3BP1-VCP complex further promotes the formation of the G3BP1-VCP-FAF2 complex, which is essential for the SG disassembly at the endoplasmic reticulum membrane [106]. VCP is also required for SG clearance involving proteasomal degradation of ubiquitinated proteins [ 107, 108] . Upon arsenite treatment, ZFAND1, a UBL/zinc-finger protein, is necessary to recruit both VCP and 26S proteasome at SGs, which promotes the clearance of SGs through the proteasomal degradation pathway [107]. Deletion of ZFAND1 causes abnormal accumulation of ubiquitinated proteins, which can be eventually cleared through autophagy [107]. The clearance of aberrant SGs through autophagy is significantly slower than the proteasomal clearance of the SGs. Together, particular Ub modifications and polyUb chains are involved in the disassembly and clearance of normal and aberrant SGs.

Besides the readers and binders of Ub code, the erasers of Ub modifications have also been found to be involved in regulating SG dynamics. In one study, the K48- and K63-linked polyUb chains and two DUBs, USP5 and USP13, were found preferentially recruited to the SGs, upon the treatment of heat shock, puromycin, or VER-155008 [87]. Depleting USP5 or USP13 with siRNAs was shown to increase the ubiquitinated protein levels, elevate the SG assembly, and repress SG clearance in response to heat shock [87]. Further experiments showed that the DUB activities of USP5 and USP13 are essential to SG clearance. Namely, the release of Ub monomers from ubiquitinated substrates is required for SG clearance because USP13 and USP5 have preferences for different Ub substrates. The two DUBs have different preferences for Ub substrates: USP13 generally hydrolyzes protein-conjugated polyUb chains, whereas USP5 hydrolyzes unanchored polyUb chains [ 87, 109– 113] . General discussion of ubiquitination in regulating SG dynamics can also be found in published reviews [ 7, 94, 114] .

### Modulation of signaling pathways

**Figure 3:**
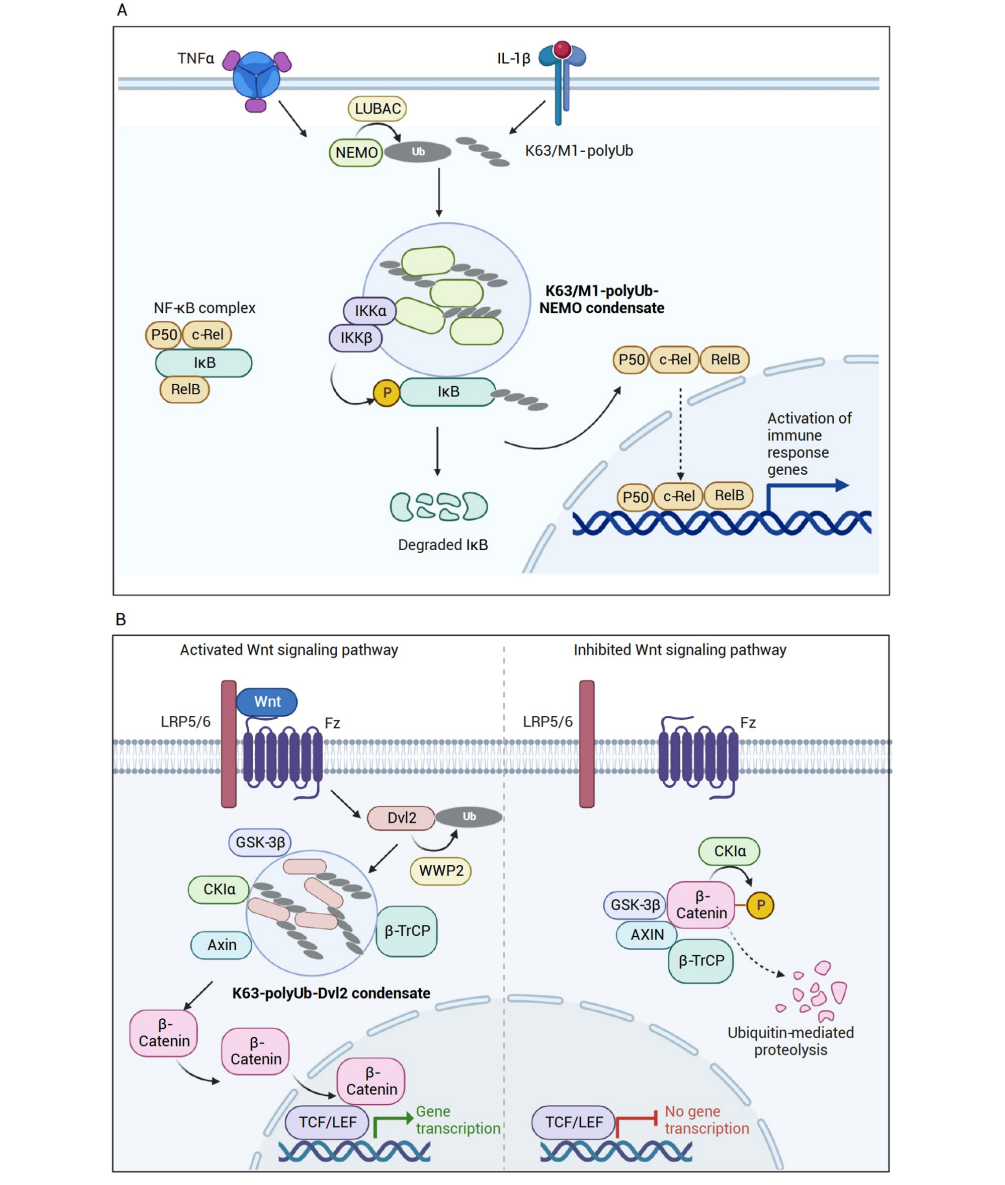
Figure 3 A schematic diagram showing how ubiquitination can participate in NF-κΒ and Wnt signaling and modulate their phase separation behavior (A) NF-κB complex is responsible for activating many downstream proteins involved in immune and inflammatory responses. NEMO condensates are formed through the non-covalent interactions between anchored polyUb chains and NEMO following the stimulation of TNFα or IL-1β. IKKα and IKKβ are further recruited to the NEMO-polyUb condensates, which leads to the phosphorylation of IκB. Phosphorated IκB is then degraded by the ubiquitin-proteasome system, allowing for the release of NF-κB protein. (B) β-Catenin, a co-activator of transcription factors, is maintained at low levels in the cytoplasm through proteolysis mediated by the GSK3β-AXIN-βTrCP complex. When Wnt proteins bind to LRP5/6 and Fz proteins, Dvl2 can be ubiquitinated by E3 ligases WWP2, which leads to the formation of K63-polyUb-Dvl2 condensate. GSK-3β, AXIN, and βTrCP proteins may also be recruited to the condensate, preventing β-catenin from degradation and allowing β-catenin to be transferred into the nucleus for transcription activation. Generated with BioRender.com.

Ubiquitinated substrate proteins and unanchored polyUb chains may function as critical components of interaction platforms on which signaling pathways are activated ( Figure 3). For example, the nuclear factor Kappa B (NF-κB) signaling pathway regulates a multitude of cellular processes, including immune response, inflammation response, cell proliferation, and cell death [ 115– 118] . The NF-κB family is a transcription factor consisting of several proteins, including RelA (p50), RelB, and c-Rel [ 117, 119] . NF-κB is generally repressed in the cytoplasm by IκB. The repression can be relieved when IκB becomes phosphorylated by the IκB kinase complex (IKK), which contains IKKα, IKKβ, and IKKγ (also known as NEMO). The phosphorylated IκB is subsequently polyubiquitinated and degraded, resulting in the translocation of NF-κB to the nucleus, where it activates the expression of many downstream proteins. During this process, polyUb noncovalently binds or is conjugated to NEMO, which is essential for the activation of IKK [ 120– 123] . Recent studies suggested that polyUb activates IKK and NF-κB signaling through a phase-separation mechanism [ 62, 63] . After the administration of IL-1β or TNFα, NEMO and K63-polyUb are colocalized in cytoplasmic puncta exhibiting liquid-like properties. Further colocalization experiments identified several E3 ligases in these puncta [63]. HOIL-1 interacting protein (HOIP), a catalytic component in the linear polyUb chain assembly complex (LUBAC), is also recruited to the puncta. Interestingly, the cellular inhibitor of apoptosis 1 (cIAP1) and TNF receptor-associated factor 5 (TRAF5) were only found in the puncta when stimulated by TNFα but not by IL-1β. TRAF5 and cIAP1 are E3 ligases catalyzing the K63-linked polyubiquitination, while LUBAC is the only known E3 ligase for the conjugation of linear M1-linked polyUb chain [ 54, 55] . These findings indicate the context-dependent existence of K63- and M1-linked polyUb in the NEMO puncta. Notably, the authors showed that IKK is activated within the NEMO condensates [63]. On the other hand, overexpression of two DUBs, CYLD and A20, the well-known inhibitor of NF-κB, significantly reduces the number of NEMO puncta and inhibits the IKK activation. Notably, a similar finding showed that binding to polyUb chains is required but not sufficient to induce the NEMO phase separation, as NEMO N-terminal disordered region also contributes to the phase separation of NEMO [62].

The activation of the Wnt signaling pathway also undergoes a phase-separation process, which is likely regulated by ubiquitination. Wnt signaling is an evolutionarily conserved signal transduction pathway involved in cell differentiation, cell migration, cell polarity, and neurogenesis [ 124– 127] . The canonical Wnt pathway is activated upon the accumulation and translocation of β-catenin, a transcriptional co-activator, into the nucleus. In the absence of Wnt stimulus, the cytoplasmic levels of β-catenin are kept low through the Ub-dependent proteasomal degradation, which is promoted by a multiprotein ″destruction complex″, including the tumor suppressor AXIN, glycogen synthase kinase-3β (GSK-3β), casein kinase 1α (CK1α), protein phosphatase 2A (PP2A), and Ub E3 ligase βPTrCP [128]. When the Wnt protein binds to its transmembrane receptor Frizzled (Fz) and co-receptor LRP5/6, the formation of the Wnt-Fz-LRP5/6 complex triggers the phosphorylation of LRP6 by GSK-3β and the recruitment of AXIN. This protein complex further recruits and activates the cytoplasmic scaffold protein Disheveled (Dvl). Activated Dvl disassembles the “destruction complex” and prevents β-catenin from degradation [125]. The accumulated β-catenin can translocate to the nucleus and serves as a co-activator for the expression of Wnt downstream genes [ 129, 130] . Dvl plays a critical role in Wnt signaling [131], and several studies indicated that the phase separation of Dvl is required for the Wnt signaling activation [ 132– 137] . A recent study further suggested that the phase separation of Dvl2 is also ubiquitination-dependent [64]. The depletion or mutation of WWP2, an E3 ligase required for Dvl2 ubiquitination, leads to defective Dvl2 condensate. Furthermore, the authors suggested that the covalent modification of Dvl2 with K63-linked polyUb is essential to the formation of Dvl2 condensates, as the condensate formation was diminished upon introducing Ub K63R mutant or DUBs specific for K63 linkages [ 63, 65, 97] . As such, ubiquitinated proteins and possibly ubiquitin moieties can directly participate in the phase separation processes and modulate signaling pathways.

### Ubiquitination and phase separation in protein quality control pathways

**Figure 4:**
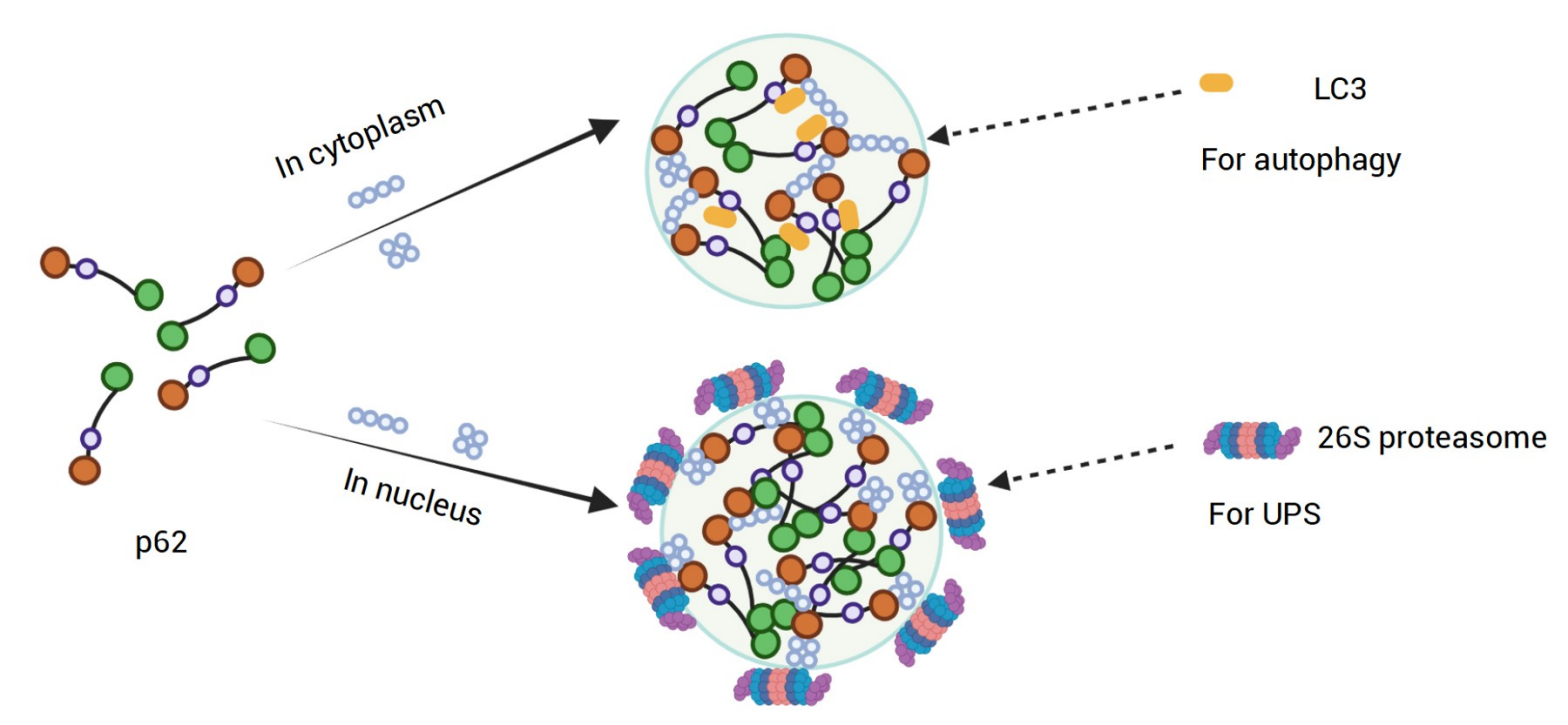
Figure 4 A schematic diagram showing how ubiquitination is essential for p62-mediated protein quality control The p62 and polyUb undergo co-phase separation; the resulting p62 bodies exist in both cytoplasm and nucleus, albeit with different architectures and functions. Cytoplasmic p62 bodies, consisting of K63-polyUb (predominantly), K48-polyUb, and LC3, are essential to autophagy; the nuclear p62 bodies, containing K48-polyUb (mostly), K63-polyUb, and certain Ub-conjugate enzymes in the interior, as well 26S proteasome at the outside, are important for protein degradation through UPS. Generated with BioRender.com.

Ub-proteasome system (UPS) and autophagy are the two major interconnected protein degradation pathways in protein quality control (PQC) [138]. Recent studies showed that phase separation leads to the formation of protein condensates specialized in PQC activities, such as proteasome-enriched foci and autophagosome precursors, in which polyUb plays essential roles [ 65, 139– 141] ( Figure 4).

The p62, also known as sequestosome-1 (SQSTM-1), is a component of various inclusion bodies in age-related and neurodegenerative cells [ 65, 142, 143] . Liquid-like p62 bodies are also found in the cytoplasm and nucleus [ 67, 138] . *In vitro*, p62 proteins form filaments and undergo phase separation in the presence of K63-polyUb [ 65, 144] . Both K48 and K63 polyUb chains exist in the formed p62 droplets, while the droplets cannot form in the USP5-treated p62 ^-/-^ cytosol, indicating that polyUb is required for the formation of p62 bodies [65]. The p62 droplets in the cytoplasm also contain LC3, an autophagosome marker, which suggests the role of p62 bodies in autophagy [65]. In addition, Ser/Thr kinase TANK-binding kinase (TBK1) has been found in the polyUb-p62 condensate, which may function as a regulator of selective autophagy [145].

The phase separation of nuclear p62 is also mediated by ubiquitinated proteins [67]. This concept is supported by the E1 inhibition experiment, in which the E1 inhibitor MLN-7243 could abrogate the generation of Ub conjugates and the formation of nuclear p62 bodies. Further studies demonstrated that p62 condensates in the nucleus contain both K48- and K63-linked polyUb. The recovery of nuclear p62 foci is much faster than that in the cytoplasmic ones when visualized with fluorescence recovery after photobleaching (FRAP) [67], suggesting that nuclear p62 bodies are more liquid-like. Further studies indicated that the nuclear p62 bodies adopt a shell-like structure, in which the outer shell is decorated with active 26S proteasome, while the inner shell mainly contains ubiquitinated proteins and Ub-conjugating enzymes. Thus, the nuclear p62 bodies may serve as proteolytic centers by sequestering the active proteasome and substrates.

Besides nuclear p62 bodies, there are reports of other proteasome-containing bodies in the cell [ 61, 67] . The sizes of these bodies are smaller than those of the p62 bodies, and they can merge with the latter [67]. The formation of these foci is p62-independent but still initiated by ubiquitination, mainly K48-linked chains [61]. In one report, the condensates comprise ubiquitinated proteins, proteasomal shuttling factor RAD23B, and 26S proteasomes [61]. The potential role of proteasome foci is to facilitate proteasomal degradation, while a more detailed discussion about the role of ubiquitination in PQC can be found in a previous review [139]. A recent study demonstrated that proteasomal degradation can be promoted by binding to and deubiquitinating branched polyUb chains, mainly K6/K48- or K11/K48-linked polyUb. The mutations of UCH37, a DUB with unique debranching activity, lead to the formation of aberrant proteasome foci [146].

## Principles Governing Ub-Modulated Phase Separation

### Multivalent interactions in Ub-modulated phase-separating systems

Phase-separating proteins interact with one other in the condensate with multivalent interactions, which can be described with a “sticker-and-spacer” model [ 60, 147, 148] . Current research has mostly focused on the phase separation behavior of intrinsically disordered proteins, in which short linear interacting motifs (SLIMs) are the stickers while the intervening flexible spacers allow for large rotational freedom of the stickers. The interactions between these SLIMs are often weaker than those between folded domains [149]. The poly-PRM (proline-rich motif)/polySH _3_ (SRC homology 3 domain), poly-SUMO/poly-SIM (SUMO interaction motif), and poly-FKBP (FK506-binding protein)/poly-FRB (FKBP-rapamycin binding domain) are the examples of phase-separation mediated by proteins comprising of multiple folded domains [ 147, 149, 150] .

Ub noncovalently interacts with other proteins by utilizing multiple interfaces, which include the I44 hydrophobic patch (usually comprising residues I44, L8, H68, and V70), the I36 hydrophobic patch (comprising residues I36, L71, and L73), and the F4 patch (comprising residues F4, Q2, and T12) [ 31, 32] . Ub also weakly interacts with another Ub noncovalently, with the phenomenological *K*_D_ value of only ~5 mM. In the ensemble structure of the Ub noncovalent dimer visualized with paramagnetic NMR spectroscopy, the two Ub monomers adopt a multitude of relative orientations, and the interactions mainly utilize the I44 patch and, to a lesser extent, the I36 and F4 patches [59]. In a polyUb chain, some of the patches may be occluded due to steric hindrance. The situation is compounded by the fact that the spacer between Ub subunits is shorter than in those well-established systems [ 147, 149] . As a result, the availability of certain “stickers” is diminished ( Figure 1A,C). In other words, the various Ub covalent linkages result in the preferential arrangement of the neighboring Ub subunits, which in turn dictates how Ub noncovalently interacts with one another.

It has been shown that polyUb chains with different linkage types and chain lengths exist in different conformations [ 151– 155] . The di-ubiquitin (diUb) is the minimal structural and possibly functional unit for a polyUb. M1-diUb and K63-diUb adopt the most extended conformation, with slight interactions between two Ub moieties except for residues that are immediately adjacent to the covalent linkage [38]. K48-diUb adopts the most compact conformation among all Ub linkages, and the conformation is stabilized by extensive interactions between two canonical hydrophobic patches (I44-patch) in each Ub unit [ 34, 156] . Such a closed conformation largely precludes the protein from interacting with other proteins. Indeed, the K48-linked polyUb chain is less likely to entangle and fibrillate upon heating or sheering than the K63-linked polyUb chain or M1-linked polyUb chain [157]. On the other hand, K11-diUb exhibits a mostly compact conformation, though the driving force appears entirely polar [37].

The conformational diversity of polyUb increases with the chain length and complexity of the chain types, i.e., mixed or branched linkages ( Figure 1C). Moreover, various physiological conditions have been shown to modulate the preferred relative arrangement of the Ub subunits in a polyUb chain [ 152, 153] . Such structural plasticity would allow the polyUb chain to recognize its target proteins specifically, and would also make the polyUb exhibit different phase separation behavior either by itself or with other proteins.

### Scaffold-client co-phase separation

**Figure 5:**
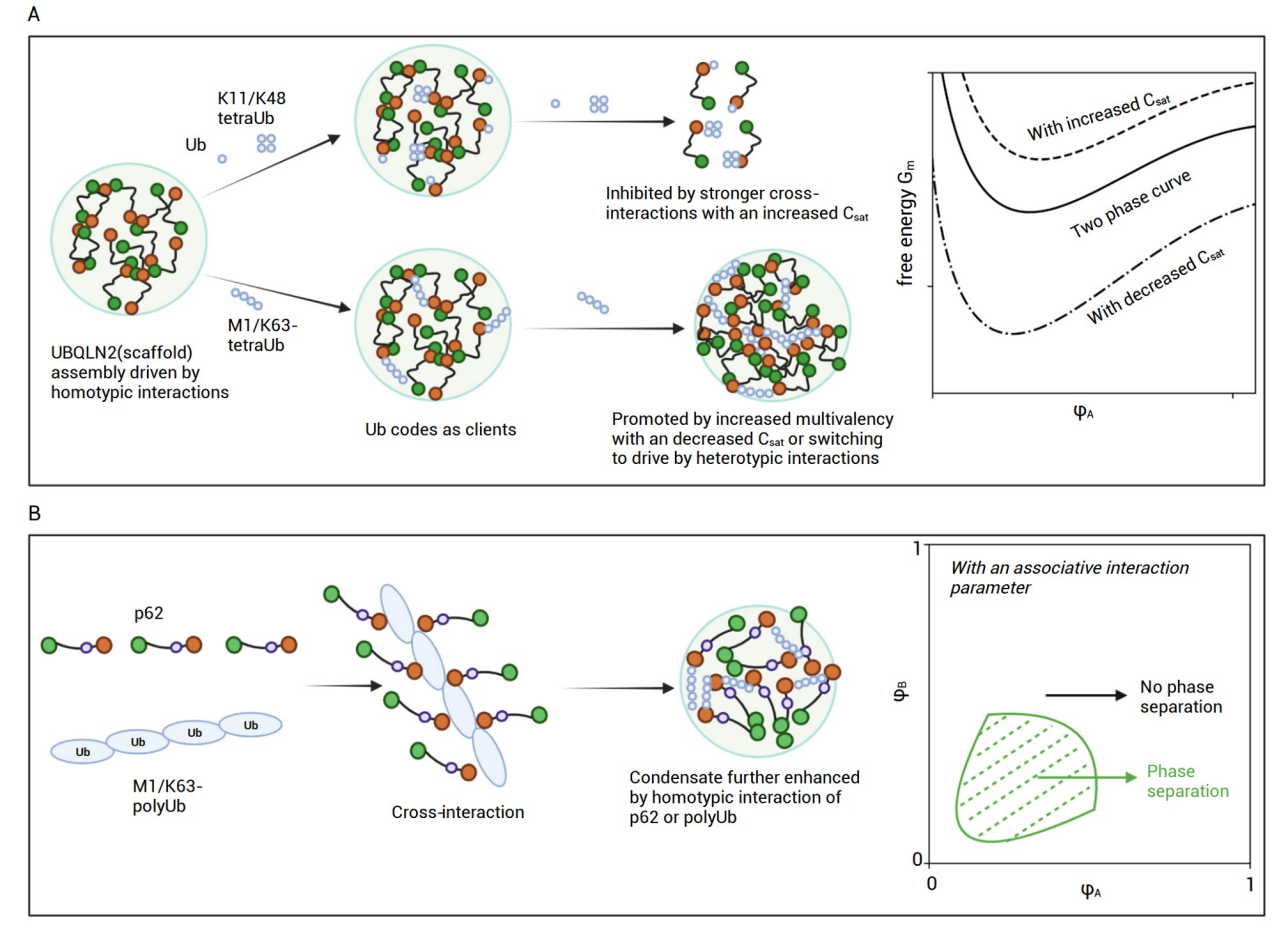
Figure 5 A proposed unifying model of ubiquitination in regulating phase separation (A) Schematic representation of Ub modification in the scaffold-client co-phase separation. Taking the UBQLN2 system as an example, its phase separation is mainly driven by the homotypic interactions between UBQLN2 proteins. The presence of Ub monomer or polyUb chains that preferentially adopt compact conformations, such as K11- and K48- tetraUb, would disrupt the UBQLN2 oligomerization owing to the strong interaction between Ub and UBA domain of UBQLN2, which leads to an increased C sat (dashed line) and inhibits phase separation. In contrast, in the presence of polyUb chains that preferentially adopt extended conformations, the C sat is likely decreased (dash-dotted line). (B) A schematic representation that Ub modification promotes phase separation. In the case of p62 bodies, the multivalent interaction between polyUb and p62 allows the phase separation to occur (in this case, the system obtains a large enough associative interaction parameter). Here, neither component alone can phase separate, and they both have to reach a threshold concentration—depending on the phenomenological binding affinity—at a particular cross-section. For more physicochemical details of the cross-interaction-driven co-phase separation, please refer to references [ 161, 162] . Generated with BioRender.com.

Scaffold-client co-phase separation is defined as the system containing one component capable of undergoing phase separation, while other components interact with the first one [20]. An example of such a system is the UBQLN2 condensate, in which UBQLN2 serves as the scaffold molecule and drives the phase separation, while the Ub serves as a client recruited to the condensed phase ( Figure 5A) [ 97, 158] . UBQLN2 contains an N-terminal folded UBL domain, two STI1 domains, a PXX domain (proline-rich domain), and a C-terminal UBA domain, all connected by flexible linkers. Biophysical characterizations indicated that the second STI1 domain primarily drives the UBQLN2 polymerization and phase separation, while the phase separation is also regulated by other domains [ 97, 159, 160] . The addition of Ub monomer can diminish the phase separation of UBQLN2, owing to the noncovalent interactions between Ub and the UBA domain of UBQLN2 ( *K*_D_ ≈ 8 μM). The modulatory effect of Ub can be explained from the threshold concentration of phase separation. Phase separation may only occur when the concentration of UBQLN2 is above a certain threshold saturation concentration ( *C*_sat_). The binding between Ub and UBQLN2 UBA reduces the effective protein concentration of the latter and in effect, increases the concentration that is required to phase separate ( Figure 5A)

Recently it has been shown that similar to the Ub monomer, K48-linked or K11-linked chains with less than four Ub units also inhibit UBQLN2 phase separation, whereas M1-linked or K63-linked polyUb chains generally promote phase separation [66]. Interestingly, longer polyUb chains were shown to promote phase separation regardless of the covalent linkage and chain length. K48-linked or K11-linked short polyUb chains mostly exhibit compact conformations. Thus, the Ub-UBQLN2 interaction would occlude the interacting surfaces on Ub and UBQLN2, therefore increasing the *C*_sat_ value ( Figure 5A). On the other hand, M1-linked, K63-linked polyUb chain, or long polyUb chains generally prefer extended conformation, and its addition enhances the effective concentration of UBQLN2 and decreases the *C*_sat_ value of UBQLN2 for phase separation. Alternatively, the addition of extended polyUb chains, at a sufficient ratio, possibly switches from homotypic interactions (between UBQLN2) and heterotypic interactions (between UBQLN2 and polyUb) in the mechanism of phase separation.

### Cross-interaction-driven co-phase separation

The p62 and polyUb are generally incapable of forming condensates independently, and may only form p62 bodies when both are present [ 20, 65, 138] . The p62 protein contains an N-terminal PB1 domain (UBL-like domain) and a C-terminal UBA domain, which are connected by a flexible linker. Several biophysical experiments, including nuclear magnetic resonance (NMR) and surface plasmon resonance (SPR), revealed that the UBA domain of p62 binds to Ub monomer, K48-diUb, and K63-diUb, with the *K*_D_ values of 540 μM, 2160 μM, and 169 μM [ 163, 164] . Moreover, from NMR titrations, at least two regions of the UBA domain were found to contribute to the Ub binding [163]. The relatively higher affinity to K63-diUb provides the structural basis for K63-polyUb-induced p62 phase separation. This concept is supported by one study in which the authors observed the p62 droplets *in vitro* by adding K63-linked polyUb, but not Ub monomer [65]. Studies have also shown that the PB1 domain of p62 promotes the oligomerization of p62 [ 138, 144, 165] . Thus, the phase separation is driven by the weak, multivalent interactions between p62 and K63-linked polyUb, and is enhanced by p62 oligomerization ( Figure 5B).

The NEMO system provides another example, in which the multivalent interactions between NEMO and K63-polyUb or M1-polyUb drive phase separation [63]. NEMO contains two polyUb binding regions: NUB and ZF domain. The ability to form a head-to-head dimer provides each NEMO protein with four binding sites for a polyUb. On the other hand, a long polyUb chain provides multiple binding sites, thus allowing multivalent interactions. Interestingly, how polyUb mediates NEMO co-phase separation does not simply depend on the binding affinity or conformational preference. The M1-diUb has stronger binding ( *K*_D_ ≈ 4.5 μM) than the Ub monomer ( *K*_D_ ≈ 152 μM), K48-diUb ( *K*_D_ ≈ 169 μM), and K63-diUb ( *K*_D_ ≈ 140 μM) from SPR analysis [166]. Yet, only K63- and M1-tetraUb were observed to induce NEMO phase separation [63].

Unlike the scaffold-client co-phase systems, there is no fixed *C*_sat_ for each component in cross-interaction-driven co-phase separating systems as neither pure component could undergo phase separation alone [ 167, 168] . Instead, specific interactions and ratios for the components are essential to the phase separation. Therefore, the appearance of the phase diagram is the determined by both interaction affinity (by extension, the Ub linkage) and the number of binding sites (chain length).

### Phase separation with more components and multiple condensed phases

In cells, the condensates contain many different molecules, making them more complex than those reconstructed *in vitro*. Though elucidating the exact mechanism of these condensates can be difficult, useful information can still be garnered by considering the strength of the homotypic and heterotypic noncovalent interactions between the components. In addition, a general model of complex biological condensates has emerged that in the biological mixtures, a small number of molecules may preferentially undergo de-mixing [169]. In such multiple-component systems, polyUb may function as a multiple-modular binding platform to increase the local effect concentrations and to facilitate the multivalent interactions, e.g., K63-polyUb-dependent formation of G3BP1-VCP-FAF2 complex [106].

Some cellular condensates and MLOs are characterized by more complex morphologies, such as multi-layered core-shell structures within large condensates. For example, the nuclear p62 body has an inner shell consisting of ubiquitinated proteins and Ub-related enzymes, and an outer shell decorated with 26S proteasome ( Figure 4) [67]. These structures are likely formed when several components have stronger interactions and prefer to cluster together. Such arrangements may also prevent the condensates from rapidly fusing with each other, akin to the Pickering emulsion [170].

## Conclusions and Future Perspective

Over the past decade, tremendous progress has been made to elucidate the basic principles for biomolecular phase separation. PTMs are known to modulate biomolecular phase separation [ 171– 182] . In this review, we focus on the relationship between ubiquitination and phase separation, and aim to provide a systematic perspective on the *pros* and *cons* of ubiquitination on the formation of protein condensates. Unlike other PTMs, the modifier itself is a protein itself, and through complicated Ub linkages ubiquitination provides a platform for multivalent interactions. The exact modulatory effect on phase separation, however, depends on the covalent architecture of the polyUb chains ( Table 1).

**Table:** **Table 1** Representative examples of ubiquitination for the regulation of phase separation

Incidence	Involved Ub linkage	Favor/disfavor phase separation	Reference
Arsenite, UV, mitochondrial stress, or heat-induced SGs	Not highly chain type-specific	Promotie	[103]
Heat-induced SGs	Monoubiquitin	Disassemble	[87]
UBQLN2 phase separation	Monoubiquitin	Disassemble	[97]
Arsenite-induced SGs	Not highly chain type-specific	Disassemble	[107]
Cytoplasmic p62 condensate	K63 polyUb	Promote	[65]
Nuclear p62 condensate	K48, K63 polyUb	Promote	[67]
Heat-induced SGs	Not highly chain type-specific	Disassemble	[ 99, 106]
Proteasome condensate	Monoubiquitin	Disassemble	[146]
UBQLN2 phase separation	K11, K48 polyUb	Disassemble	[66]
UBQLN2 phase separation	K63, M1 polyUb	Promote	[66]
NEMO phase separation	K63, M1 polyUb	Promote	[ 62, 63]
Dvl2 phase separation	K63 polyUb	Promote	[64]

Ub itself can be modified with other PTMs. Recent studies have shown that Ub can be phosphorylated at S65 and T66 by kinase PINK1 [ 183, 184] . The phosphorylation promotes an alternative Ub conformation, with the C-terminal tail retracted by two residues [185], causing a disruption of the I44 hydrophobic patch. The modulation of tertiary structure would further impact the relative arrangement of adjacent Ub moieties in the polyUb chains and modulate the interactions with other proteins [186]. The phosphorylation of Ub also has a general inhibitory effect on many enzymes responsible for attaching and removing Ub modifications and therefore would have a profound impact on the Ub signaling network and the formation of protein condensates [187].

Ub also exhibits a high tendency to assemble in the presence of metal ions with high concentrations. Biophysical experiments revealed that Hg ^2+^, Cd ^2+^, Pt ^2+^, or Zn ^2+^ could bridge residues from two or more Ub molecules, leading to the formation of protein clusters [ 188– 191] . Intriguingly, the relevance of Zn ^2+^-mediated Ub aggregation is supported based on the detection of high zinc ions concentrations in neurons, which is related to the onset of Alzheimer’s, Parkinson’s diseases, and other neurodegenerations [ 192– 194] . Thus, metal-Ub interactions add another layer of complexity to the regulatory effect of ubiquitination on protein condensates, which awaits further exploration.

Finally, phase separation in the cell by no means reaches a thermodynamic equilibrium. Ubiquitination provides a multivalent interaction platform and an intracellular spatial organization hub for dynamic signaling. But unlike other modifications, Ub itself is a protein, and therefore long and complex polyUb chains can cause macromolecular crowding, restrict free diffusion, and modulate protein interaction kinetics. The research of Ub-modulated phase separation is still a fast-growing research area until a more unified and physiochemical understanding can be reached.
